# Kinetic Analysis of Methane Hydrate Formation with Butterfly Turbine Impellers

**DOI:** 10.3390/molecules27144388

**Published:** 2022-07-08

**Authors:** Sotirios Nik. Longinos, Dionisia Dimitra Longinou, Nurbala Myrzakhmetova, Nazgul Akimbayeva, Mariamkul Zhursumbaeva, Kaldibek Abdiyev, Zhexenbek Toktarbay, Mahmut Parlaktuna

**Affiliations:** 1 Department of Petroleum Engineering, Nazarbayev University, Nur-Sultan 010000, Kazakhstan; 2 Department of Petroleum and Natural Gas Engineering, Middle East Technical University, Ankara 06800, Turkey; mahmut@metu.edu.tr; 3 Department of Economics & Sustainable Development, Harokopio University, 17676 Athens, Greece; dlogginou@gmail.com; 4 Department of Chemistry, Faculty of Natural Science, Kazakh National Woman’s Teacher Training University, Almaty 700420, Kazakhstan; myrzakhmetova.nurbala@qyzpu.edu.kz (N.M.); akimbayeva73@qyzpu.edu.kz (N.A.); 5 Department Chemical Processes and Industrial Ecology, Satbayev University, Almaty 050013, Kazakhstan; m.zhursumbayeva@satbayev.university (M.Z.); k.abdiyev@satbayev.university (K.A.); 6 Advanced Solar Energy Materials and System Lab, National Laboratory Astana (NLA), Nazarbayev University, Nur-Sultan 010000, Kazakhstan

**Keywords:** gas hydrates, hydrate formation, butterfly turbine, induction time

## Abstract

Heat generation during gas hydrate formation is an important problem because it reduces the amount of water and gas that become gas hydrates. In this research work, we present a new design of an impeller to be used for hydrate formation and to overcome this concern by following the hydrodynamic literature. CH_4_ hydrate formation experiments were performed in a 5.7 L continuously stirred tank reactor using a butterfly turbine (BT) impeller with no baffle (NB), full baffle (FB), half baffle (HB), and surface baffle (SB) under mixed flow conditions. Four experiments were conducted separately using single and dual impellers. In addition to the estimated induction time, the rate of hydrate formation, hydrate productivity and hydrate formation rate, constant for a maximum of 3 h, were calculated. The induction time was less for both single and dual-impeller experiments that used full baffle for less than 3 min and more than 1 h for all other experiments. In an experiment with a single impeller, a surface baffle yielded higher hydrate growth with a value of 42 × 10^−8^ mol/s, while in an experiment with dual impellers, a half baffle generated higher hydrate growth with a value of 28.8 × 10^−8^ mol/s. Both single and dual impellers achieved the highest values for the hydrate formation rates that were constant in the full-baffle experiments.

## 1. Introduction

Natural gas hydrates, frequently called clathrates, are crystalline compounds. The formation of clathrate hydrates occurs due to host water molecules covering guest gas molecules such as methane, ethane, propane, isobutene, n-butane, nitrogen, carbon dioxide, and hydrogen sulfide [1]. There are three alternative structures of hydrates: structure I (sI), structure II (sII), and structure H (sH), which are separated based on the type and number of cavities, as well as the magnitude of the gas molecules [2]. The formation of hydrates is an exothermic crystallization process that is defined by nucleation followed by size enlargement and an accumulation of crystals. Under standard conditions of temperature and pressure, methane hydrates can contain 150–180 *v*/*v*, hence they indicate significant gas storage properties [3,4]. The gas hydrates topic has attracted the interest of many researchers from different fields due to its application as an alternative energy resource, which is suitable for gas storage technology and gas transportation, along with separation technology and CO_2_ sequestration [5,6,7,8].

The practice in the industry of hydrates as discussed has been an arguable matter in the field of engineering. A few of the initial drawbacks are the inactive creation, unreacted median water as a great percentage of the hydrated mass, and the credibility of hydrate storage ability and economy of process scale-up [9]. The stirring style is the optimum way to ameliorate heat and mass transfer in methane hydration implementation: induction time is abbreviated, formation rates are accelerated, and storage capacity is augmented when stirring is applied. Stirring reactors are extensively implemented in research works on the formation and dissociation of gas hydrates [10,11,12]. Apart from the reactors, flow hence different blades with or without baffles play a crucial role and they can affect gas hydrate formation. Previous research groups that investigated kinetic analysis of different flows and different gases at different pressure and temperature conditions indicated that radial flow is better compared with mixed flow for both single and dual-impeller experiments [13,14,15].

There are some research works examining hydrodynamic behavior along with gas hydrate formation. Douieb et al. (2015) investigated three alternative impellers such as the dispermax, pitched-blade turbine downward pumping (pbtu), and maxblend with only full baffles for CO_2_ gas hydrate formation. Their results showed that maxblend has the best conductivity in energy performance, followed by dispermax and then pbtu [16]. Longinos and Parlaktuna examined Rushton turbine (rt), pitched-blade turbine upward pumping (pbtu), and pbtd with no baffles, full baffles, half baffle, and surface baffles for both single and dual impellers. Their outcomes revealed that for both methane and methane–propane gas hydrate formation, the Rushton turbine had better behavior in both induction time and rate of hydrate formation. The reason was that the Rushton turbine presented better pumping capacity, uniform shear field, and good contact ability between gas and liquid. Experiments with surface baffle displayed higher values in the rate of hydrate formation, indicating that vortex may play a positive role in hydrate formation, under specific conditions such as height and width. Generally, baffle played a positive role in gas hydrate formation compared with experiments with no baffles [4,14,15]. This work discusses the design and evolution of a continuously stirred tank reactor, with an inner volume of 5.7 L with an experimental approach and not by a modeling approach [17,18,19]. One of the main problems that previous research groups faced was the generated heat because hydrate formation is an exothermic reaction. The intention and novelty of this research work was to design a new impeller that would form hydrates for more than three hours. This new type of impeller would form gas hydrates with alternative types or no baffles following hydrodynamic designs, for a single impeller the volume of water is 2.65 L and for dual impellers the volume is 3.8 L.

## 2. Results and Discussion

The outcomes of dual and single impellers are presented in Table 1. Stirring started at a pressure of 42.5 bars and a temperature of 2 °C.

Analysis of BT experiments indicates, unlike the first nuclei periods, a reliance on the category of baffle used. The shortest first nuclei period was 2 min with full baffles while it was 1 h and 20 min and 1 h and 21 min for half and surface baffles, respectively. In a no-baffle case, it took 10 h and 36 min to notice the outset of hydrate formation. Due to a high induction time for BT with no baffles, this test was conducted and a first nuclei period of 11 h and 14 min was acquired (the rate of hydrate formation was almost the same in both experiments). It should not be forgotten that the process of hydrate formation is a stochastic process. Baffles are impeding perpendicular fixed planes or extended plates to pause radial swirl and transform rotary to axial flow. When the length of baffles covers the total length of a reactor (full baffle), axial movement is anticipated. When the length of baffles is smaller such as in occasions of half or surface baffles, tangential movement is prevalent at the lowest part of the reactor [4,14,15].

When baffles are absent, tangential flow is prevalent everywhere, and a middle swirl is composed. The middle swirl could be beneficial in embodying gas if the swirl is extended in the impeller, but an immoderate amount of gas would have a considerable action on the hydrodynamics in the vessel and the gas quantity because a vortex can create an unwelcome status [20]. The use of full baffles forms competent gas entrance access to the system and the axial flow that is created by baffles assists with the formation of the first nuclei of gas hydrates; hence induction time starts more rapidly [21]. As the length of baffles gets shorter, the potential disconnect between the lower part of the tank and the upper part, where baffles are present, does not permit for such competent gas–liquid contact and induction time augments [4,15].

**Figure 1:**
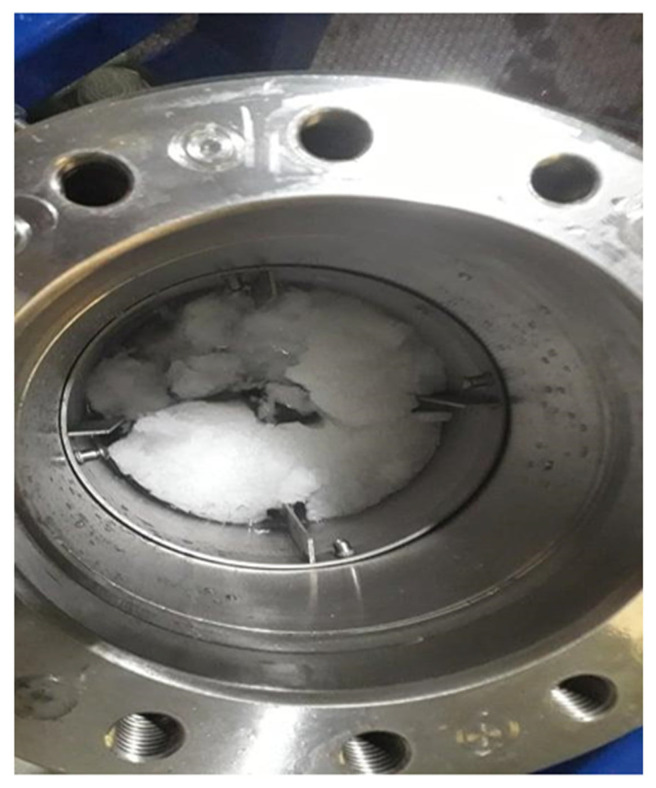
Methane hydrate formed with single impeller and surface baffle.

As it can be seen from Table 1, hydrate yield for all experiments is not big for two main reasons: firstly, because the total duration of experiments was only 3 h after hydrate formation, and secondly, due to the high quantity of water. In these experiments, the hydrate formation followed the hydrodynamic literature [20,21] about baffles and water quantity, this means that the height of water could be either equal to or 1.2 times the inside of the reactor’s diameter for single impellers, and 1.2–1.6 times for dual-impeller experiments. Figure 1 shows the amount of gas hydrate in the reactor for a single-impeller experiment with surface baffle.

**Figure 2:**
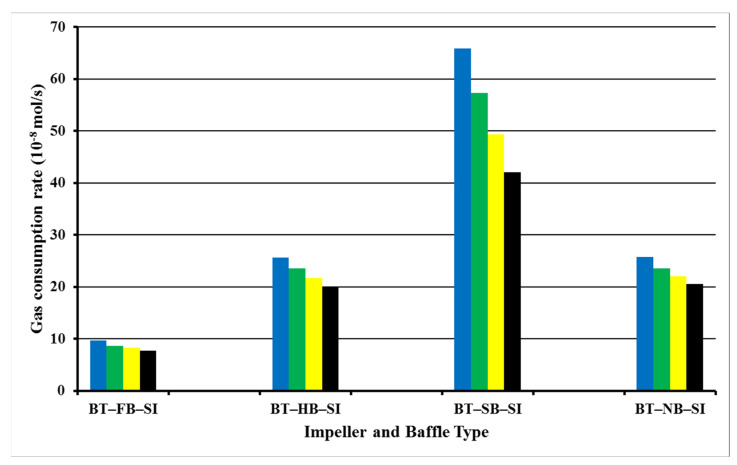
Rate of gas consumption or equivalent hydrate formation in single-impeller experiments at starting rotation conditions of 2 °C and 42.5 bars for 1 s (blue), 600 s (green), 1200 s (yellow), and 1800 s (black).

Column 3 in Table 1 presents the period of hydrate formation for our experiments. It is obvious that it was potent to create hydrates for more than 3 h for all BT experiments. The main reason for not having a shortened hydrate formation period in BT experiments is the lower augmentation in temperature following hydrate formation. In all experiments, an augmentation in the temperature of the system is observed with the formation of the first nuclei of gas hydrates since the reaction is exothermic. The change in the number of moles of free gas after the initiation of hydrate formation was used to calculate the rate of hydrate formation at four different times (1 s, 600 s, 120 s, and 1800 s). Figure 2 presents the hydrate formation rates of single-impeller experiments.

Hydrate formation rates of BT experiments with full baffles are always lower than hydrate formation rates of BT experiments with half, surface, or no baffle. The highest value of the rate of hydrate formation is 65.9 × 10^−8^ mol/s for surface baffle, and then comes no-baffle experiment with the value of the rate of hydrate formation as 25.8 × 10^−8^ mol/s, which is quite close to the value of half-baffle experiment, 25.6 × 10^−8^ mol/s. The experiment with a full baffle has the lowest value of the rate of hydrate formation, 9.7 × 10^−8^ mol/s. These outcomes show that the central vortex that is created due to no baffle or to less baffle (surface) functions positively in our system. These outcomes show that BT experiment with surface baffle creates a better interplay between gas and liquid in contrast to other experimental tries. Superior pumping ability, equable shear field, and good touch ability can be concluded to be the cause of this upshot [21].

**Figure 3:**
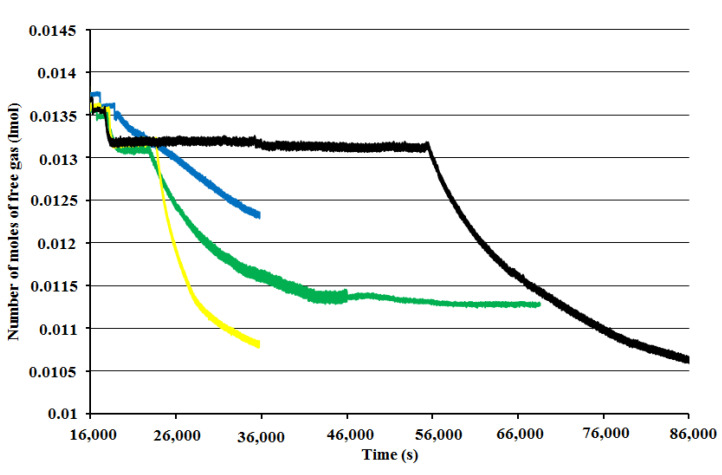
Number of moles of free gas for single-impeller experiments vs. time at starting rotation properties 2 °C and 42.5 bars for full-baffle experiment (blue), half-baffle experiment (green), surface-baffle experiment (yellow), and no-baffle experiment (black).

The hydrate formation rate of BT experiments with surface baffle decreases faster than any other of the three experiments. On the other hand, there was a small reduction in the hydrate formation rate with time for BT experiment with a full baffle which began with a relatively low hydrate formation rate. This variation actually may generate the shortening of the period of hydrate formation [4,22,23]. Figure 3 shows the number of moles of free gas with the time for single impellers.

**Figure 4:**
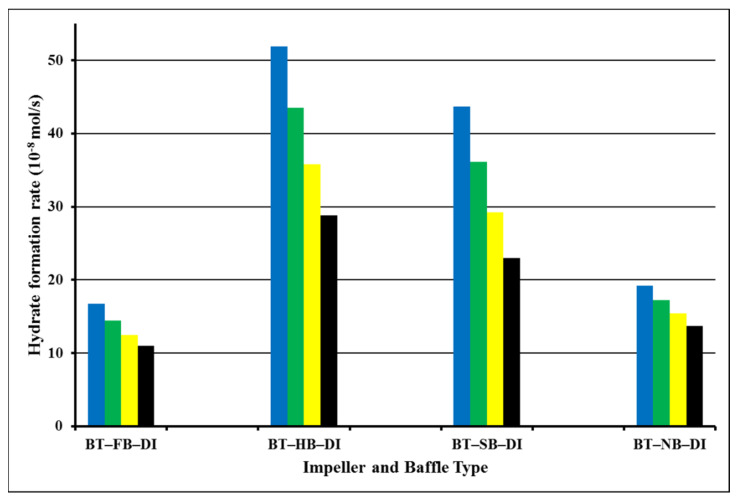
Rate of hydrate formation in dual-impeller experiments at starting rotation properties 2 °C and 42.5 bars for 1 s (blue), 600 s (green), 1200 s (yellow), and 1800 s (black).

Analysis of the data of BT/BT dual-impeller experiments for induction time shows the same trend for single-impeller experiments (Figure 4). The shortest induction time is with full baffles (FB), 2 min. Then, induction times were not augmented in identical ways such as in single-impeller experiments. In dual-impeller experiments, the longer induction time takes place in the experiment with a surface baffle (SB). Therefore, the discussion made on the effect of baffle types on the induction time is different in systems with single, and in systems with dual impellers [24,25]. Figure 4 presents the hydrate formation rates of dual-impeller experiments obtained from the analysis of Figure 3 for 1 s, 600, 1200, and 1800 s, respectively.

**Figure 5:**
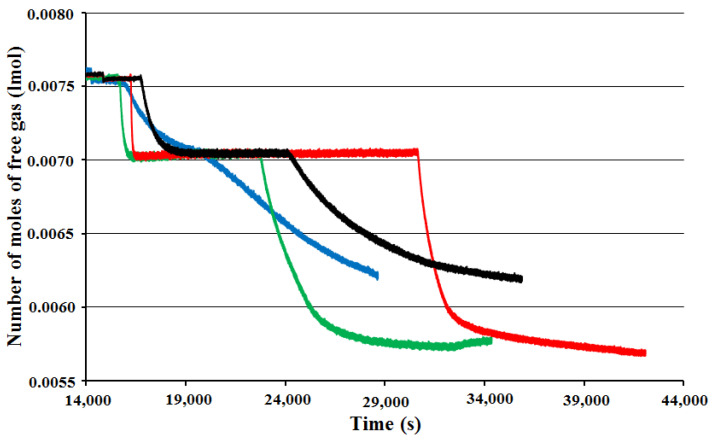
Number of moles of free gas for dual-impeller experiments vs. time at starting rotation properties 2 °C and 42.5 bars for full-baffle experiment (blue), half-baffle experiment (green), surface-baffle experiment (red), and no-baffle experiment (black).

Hydrate formation rates of BT experiments with full baffles are always lower than hydrate formation rates of BT experiments with half, surface, or no baffle, which is the same as in a single experiment. The highest value of the rate of hydrate formation was 51.9 × 10^−8^ mol/s for a full baffle, and next was the surface-baffle experiment with a value of the rate of hydrate formation at 43.7 × 10^−8^ mol/s. The experiment with a full baffle had the lowest value of the rate of hydrate formation, 16.7 × 10^−8^ mol/s, which was quite close to a no-baffle experiment value which was 19.2 × 10^−8^ mol/s. This shows that the short length of the central vortex that was created functioned positively in our system, while no vortex or a lengthy vortex function negatively. These outcomes show that BT experiments with half and surface baffle create better interactions between gas and liquid compared with the other two experiments, showing a behavior which nominates a better level of gas–liquid contact. Mass transfer impedances could be perceptibly reduced which finally leads to beneficial mixing intensity [26,27]. It should be noticed from the last column of Table 1 that the repeated (duplicated) experiments had almost the same rate of hydrate formation ranging from 1.25 to 1.92 for the first second and from 1.01 to 1.35 for 600 s, confirming our experiments in both the single- and dual-impeller experiments. Figure 5 shows the number of moles of free gas with the time for dual impellers.

**Figure 6:**
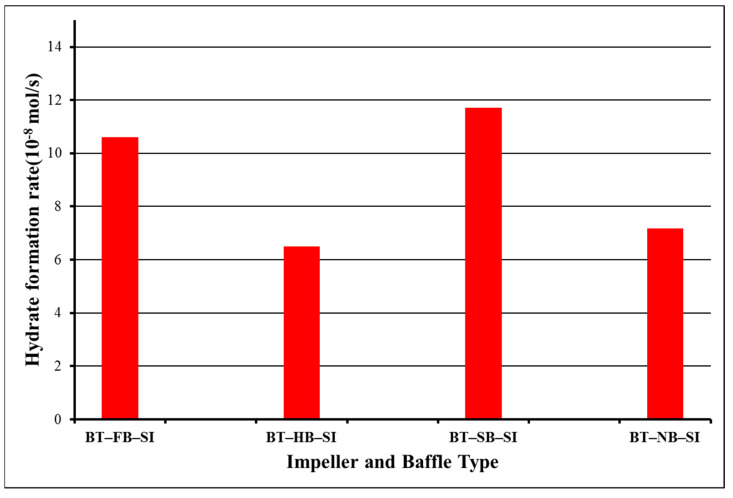
Hydrate formation kinetics values for 10,800 s in single impellers at starting rotation properties of 2 °C and 42.5 bars.

**Figure 7:**
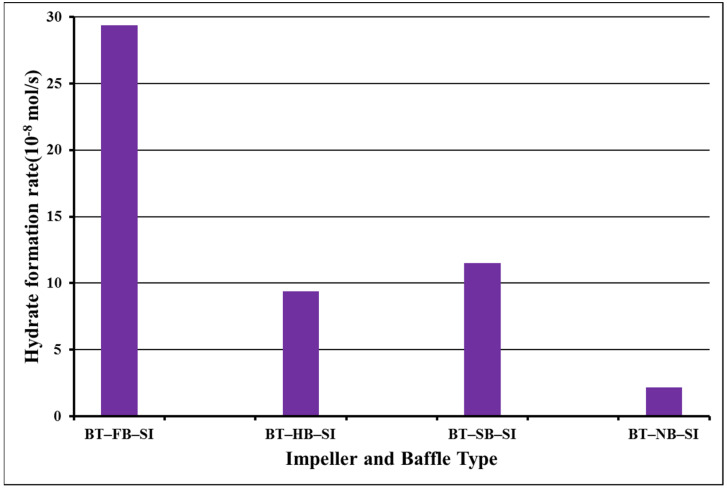
Hydrate formation kinetics values for 10,800 s in dual impellers at starting rotation properties of 2 °C and 42.5 bars.

The ultimate factor that is calculated is the hydrate formation rate constant (αΚ*). First, there is a calculation of the hydrate formation kinetics (r_hy_) for three hours in the same way that the rate of hydrate formation for 1, 600, 1200, and 1800 s were calculated. The results indicated that the values of the rate of hydrate formation for a 3 h process varied from 3.16 × 10^−8^ mol/s (surface-baffle experiment) to 1.5 × 10^−8^ mol/s (half-baffle experiment) for single-impeller experiments, and from 9.13 × 10^−8^ mol/s (surface-baffle experiment) to 1.22 × 10^−8^ mol/s (no-baffle experiment) for dual-impeller experiments. In our system, surface-baffle experiments in both single and dual impellers show the best rate of hydrate formation for the whole process. Figure 6 and Figure 7 show the hydrate formation kinetics (rate of hydrate formation) for a 3 h process.

**Figure 8:**
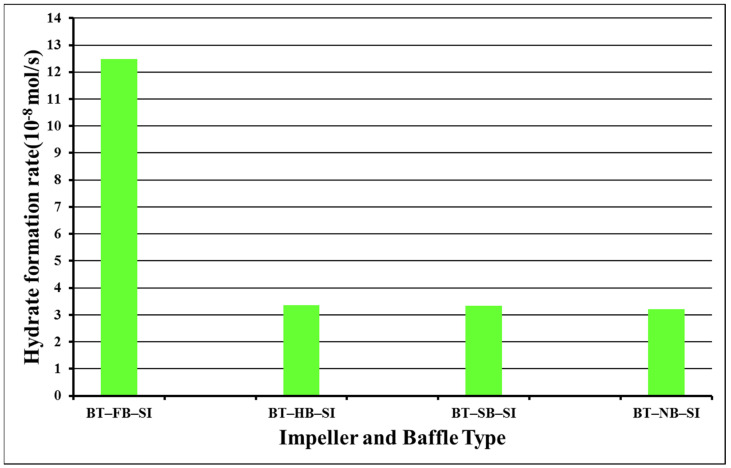
Hydrate formation rate constant in single-impeller experiments.

**Figure 9:**
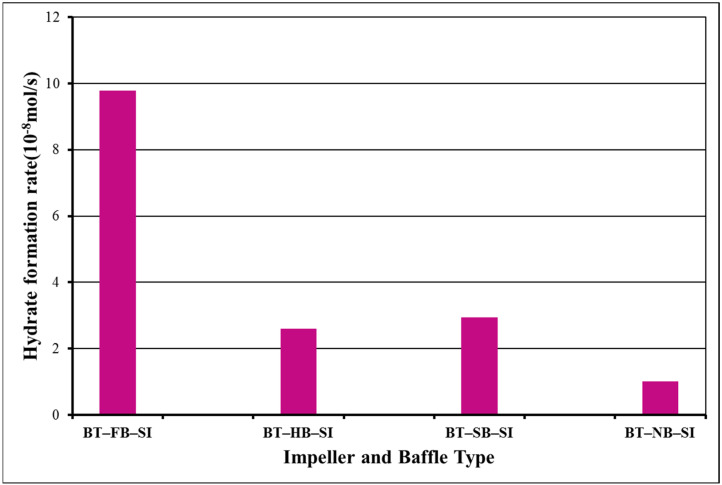
Hydrate formation rate constant in dual-impeller experiments.

The calculation of fugacity for Equation (6) can be completed using the pressure and fugacity coefficient (by the use of Peng Robinson EOS). Figure 8 and Figure 9 indicate the results of the hydrate formation rate constant (αΚ*) with the use of the COAL SEEK method for single and dual-impeller experiments, respectively.

From Figure 8 and Figure 9, the single- and dual-impeller experiments with full baffle achieved the highest αΚ* values, meaning that the rate of gas consumption for CH_4_ hydrate formation with full baffle (no central vortex through the hours after induction time) was the fastest, but does not mean that the amount of CH_4_ hydrate formed was the largest among the other three single or dual groups of experiments [28,29,30,31]. This fact takes place because with full-baffle experiments there is still time for hydrate formation (straight line and no curve which means that, in a short period, there will be hydrate stabilization and then hydrate dissociation). It should be mentioned that all calculations were conducted for 3 h after induction and not until there was a stabilization of hydrate formation, due to specific restrictions of our experimental setup.

## 3. Reactor Design

**Figure 10:**
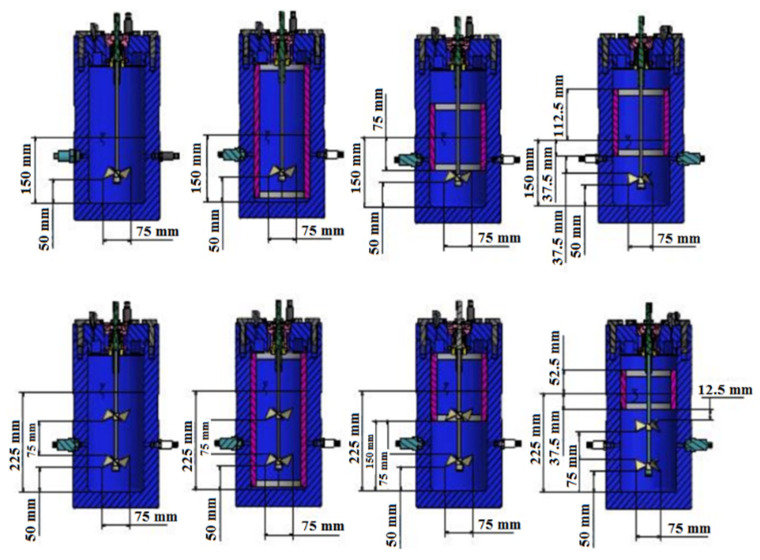
Dimensions for four single and four dual-impeller experiments with the butterfly turbine.

A continuously stirred tank reactor (CSTR) with an internal volume of 5.7 L was designed (see Supplementary Material Figure S1) for our experimental process (see previous articles of Longinos and Parlaktuna for a thorough presentation of the experimental process). The dimensions of the rector, baffles, and impellers are all explained thoroughly below in Figure 10 (see Supplementary Material Figures S2 and S3). In Figure 10, the upper row from left to right shows: a single impeller with no baffle, a single impeller with full baffle (15 cm submerged in water), a single impeller with half baffle (7.5 cm submerged in water), and a single impeller with surface baffle (3.75 cm submerged in water). The space between the two impellers in the dual one was 7.5 cm. The height of the liquid was 15 cm in the single impeller and 22.5 cm in the dual impellers. The width of baffles was 1 cm while the internal diameter of a reactor was 15 cm (see Supplementary Material Figure S4). The sparger had a height of 1 cm and was used to bring the gas in such a way that bubbles were dispersed evenly. The bottom of the clearance (distance between the bottom of the reactor and impeller) was 5 cm [4].

**Figure 11:**
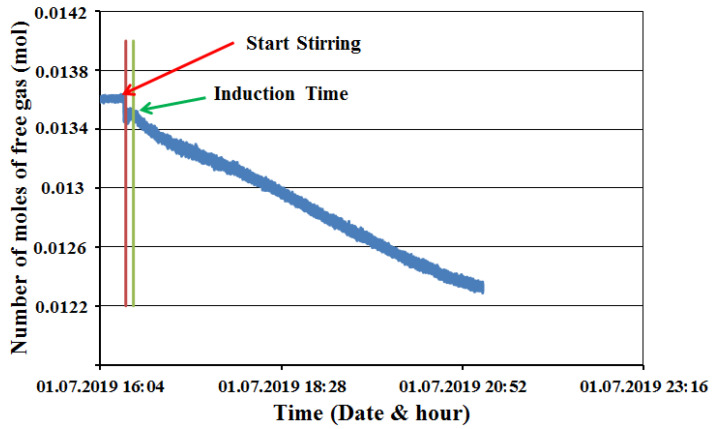
The hydrate formation process of methane single impeller full baffle with the butterfly turbine (CH_4_-SI-FB-BT).

The data collected from our experimental process are pressure, temperature, and torque for every second. The gas compressibility factor of the real gas law Z was calculated using Lee and Kesler’s (1975) compressibility factor expression [32]. A sample plot of change in the free gas number of moles is shown in Figure 11 for CH_4_-SI-BT-FB.

**Figure 12:**
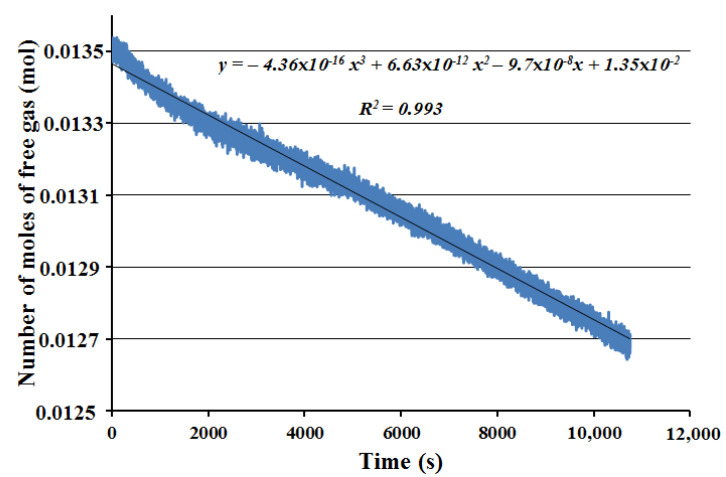
Period of the first nuclei of the hydrate formation process until the end of experimental process after 3 h.

Figure 12 was designed with the same data as Figure 11, but covering only 3 h of the hydrate formation period.

(1) $$n=-4.36\times 10^{-16}t^{3}+6.63\times 10^{-12}t^{2}-9.70\times 10^{-8}t+1.35\times 10^{-2}$$

where *n* is the number of moles of free gas, mole; and *t* is time, s.

The derivative of Equation (1) gives the gas consumption rate (Equation (2)) which is equivalently considered as the hydrate formation rate in this study.

(2) $$\frac{dn}{dt}=-3\times 4.36\times 10^{-16}t^{2}+2\times 6.63\times 10^{-12}t-9.70\times 10^{-8}$$

where: $\frac{dn}{dt}$ is the gas consumption rate, mol/s; and *t* is time, s. Table 2 presents the outcomes for rate of hydrate formation for experiment with single impeller and full baffle for 1 s, 600, 1200 and 1800 s.

The percentage of water converted to hydrate is defined from the information obtained from gas uptake and the experimental conditions using Equation (3):

(3) $$\mathrm{Conversion}\mathrm{of}\mathrm{water}\mathrm{to}\mathrm{hydrates}(\mathrm{mol}\%)=\frac{\Delta n_{H,↓}\times HydrationNumber}{n_{H_{2}O}}\times 100$$

The *hydration number* is the number of water molecules required per guest methane molecule to form hydrate, and we used the hydration number of 6.1 for calculations based on the literature [6,7]. Hydrate productivity is determined as the normalized rate of hydrate formation calculated for the first 30 min from nucleation (the start of hydrate growth), and is given by Equation (4):

(4) $$\mathrm{Hydrate}\mathrm{productivity},{\mathrm{NR}}_{30}=\frac{R_{30}}{V_{water}}(\mathrm{mol}\times L^{-1}\times s^{-1})$$

where R_30_ is the rate of hydrate growth (mol/s) for the first 30 min after the induction time.

The hydrate formation kinetics (r_hy_) for a maximum of 3 h was estimated using the chemical potential difference between formation and equilibrium as the driving force, as follows:

(5) $$r_{\mathrm{yh}}=\frac{dn}{dt}=\alpha K^{*}(\mu_{g}-\mu_{\mathrm{eq}})$$

where *n* is the number of moles the gas consumed in the supply vessel (in our occasion for a maximum of 3 h), *t* is the end of the experiment, αΚ* is the hydrate formation rate constant (α is the interfacial area, and Κ* is the overall kinetic constant), and μ_g_ and μ_eq_ are the chemical potentials of the guest molecule in the gas phase and hydrate phase, respectively. Chemical potential terms can be transformed to the fugacity of the guest molecule,

(6) $$r_{\mathrm{yh}}=\frac{dn}{dt}=\alpha K^{*}RTln(\frac{f_{g}}{f_{eq}})$$

and

*f* = *φP* (7)

where *R* is the gas constant, *T* is the temperature, and *f_g_* and *f_eq_* are the fugacity of the gas in the gas phase and hydrate phase, respectively [14,15].

## 4. Conclusions

This research work combined the kinetic and hydrodynamic behavior of the butterfly turbine impeller for methane gas hydrate formation with different or no baffles on single or dual occasions. The conclusions of our research are presented below:

- Experiments with a single impeller indicated that the butterfly turbine impeller with surface baffle has the best performance among the single-impeller experiments for the first 30 min after induction time.
- In dual-impeller experiments, half baffle and then surface baffle had the highest values of rate of hydrate formation.
- The induction time was less in both single- and dual-impeller experiments with the presence of full baffle.
- The single and dual-impeller experiment with full baffle achieved the highest formation rate constant values, meaning that the rate of gas consumption for CH_4_ hydrate formation with full baffle (no central vortex through the hours after induction time) was the fastest.

## Figures and Tables

**Table 1:** Outcomes of single and dual-impeller experiments (* where SE is the standard deviation and *n* = 2 the number of experiments while the results are for 1 and 600 s).

System	Induction Time (Hours–Minutes)	Period of Hydrate Formation (Hours)	Hydrate Growth Rate for 30 Minutes, R_30_ (10^−8^ mol/s)	Hydrate Yield/Conversion of Water to Hydrate (mol%)	Standard Error
					$*{\mathrm{SE}}_{X}=\mathrm{SE}/\sqrt{n}$
SI-BT-FB	2 min	3 h	7.74	3.35	1.25/1.01
SI-BT-HB	1 h and 20 min	3 h	19.90	5.80	1.47/1.09
SI-BT-SB	1 h and 21 min	3 h	42.0	9.75	1.35/1.28
SI-BT-NB	10 h and 36 min	3 h	20.50	5.92	1.68/1.11
DI-BT-FB	2 min	3 h	11.0	1.83	1.59/1.04
DI-BT-HB	1 h and 58 min	3 h	28.80	2.20	1.85/1.28
DI-BT-SB	4 h and 2 min	3 h	23.0	2.65	1.77/1.14
DI-BT-NB	2 h and 6 min	3 h	13.70	2.03	1.92/1.35

**Table 2:** Shows the rate of hydrate formation for 1 s, 600, 1200, and 1800 s for CH_4_-SI-PBT-FB experiment, as an example. Rates of hydrate formation CH_4_-SI-PBT-FB.

Time (s)	1	600	1200	1800
Hydrate formation rate (mole/s)	−9.7 × 10^−8^	−8.95 × 10^−8^	−8.30 × 10^−8^	−7.74 × 10^−8^

## Data Availability

The data presented in this study are available in the supplementary material.

## Supplementary Materials

The following supporting information can be downloaded at: https://www.mdpi.com/article/10.3390/molecules27144388/s1, Figure S1: Experimental set up for methane hydrate formation experiments; Figure S2: Design and dimensions of butterfly impeller; Figure S3: Butterfly impeller as was used in our experiments; Figure S4: Three different baffles used in this study.

## Sample Availability

Samples of the compounds are not available from the authors.
