# Supplementary material for: Kinetic Analysis of Methane Hydrate Formation with Butterfly Turbine Impellers
Source: Molecules. 2022 Jul 8;27(14):4388. doi: 10.3390/molecules27144388 (PMC9319823; doi:10.3390/molecules27144388)
Supplement: Supplementary file 1 [file molecules-27-04388-s001.zip › molecules-1800669-supplementary.pdf]

# Kinetic Analysis of Methane Hydrate Formation with Butterfly Turbine Impellers


**Sotirios Nik. Longinos [1,2,*], Dionisia Dimitra Longinou [3], Nurbala Myrzakhmetova [4], Nazgul Akimbayeva [4], Mariamkul Zhursumbaeva [5], Kaldibek Abdiyev [5], Zhexenbek Toktarbay [6,*] and Mahmut Parlaktuna [2]**

[1] Department of Petroleum Engineering, Nazarbayev University, Nur-Sultan 010000, Kazakhstan
[2] Department of Petroleum and Natural Gas Engineering, Middle East Technical University, Ankara 06800, Turkey; mahmut@metu.edu.tr
[3] Department of Economics & Sustainable Development, Harokopio University, Athens 17676, Greece; dlogginou@gmail.com
[4] Department of Chemistry, Faculty of Natural Science, Kazakh National Woman's Teacher Training University, Almaty 700420, Kazakhstan; myrzakhmetova.nurbala@qyzpu.edu.kz (N.M.); akimbayeva73@qyzpu.edu.kz (N.A.)
[5] Department Chemical Processes and Industrial Ecology, Satbayev University Almaty 050013, Kazakhstan; m.zhursumbayeva@satbayev.university (M.Z.); k.abdiyev@satbayev.university (K.A.)
[6] Advanced Solar Energy Materials and System Lab, National Laboratory Astana (NLA), Nazarbayev University, Nur-Sultan, 010000, Kazakhstan
* Correspondence: sotirios.longinos@nu.edu.kz (S.N.L.); zhexenbek.toktarbay@nu.edu.kz (Z.T.)


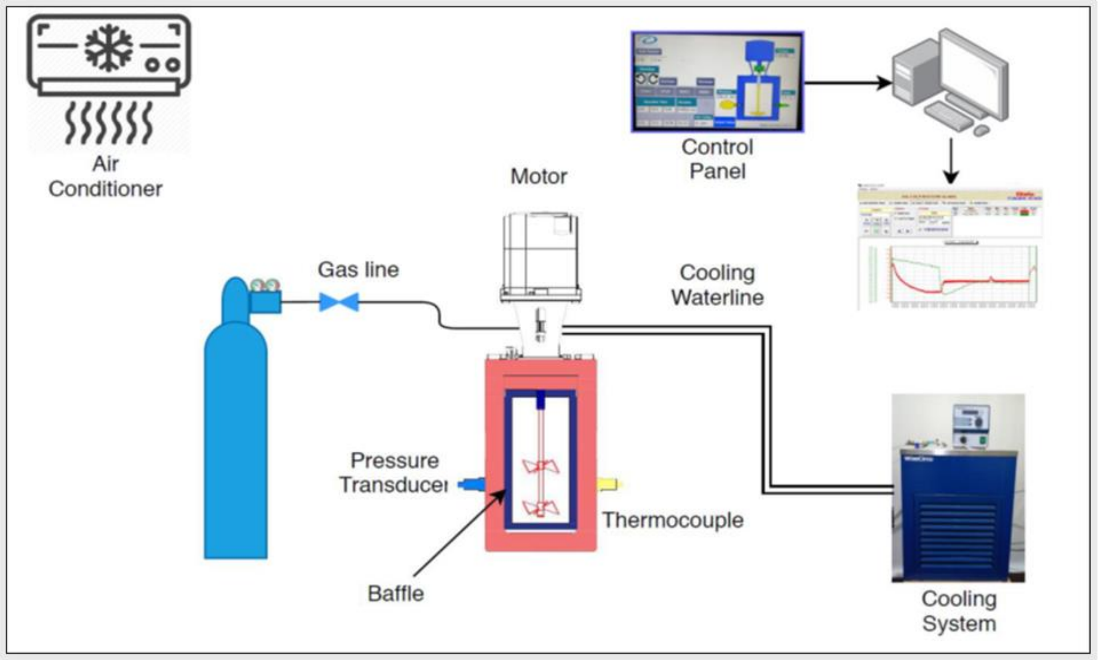


Figure S1 Experimental set up for methane hydrate formation experiments

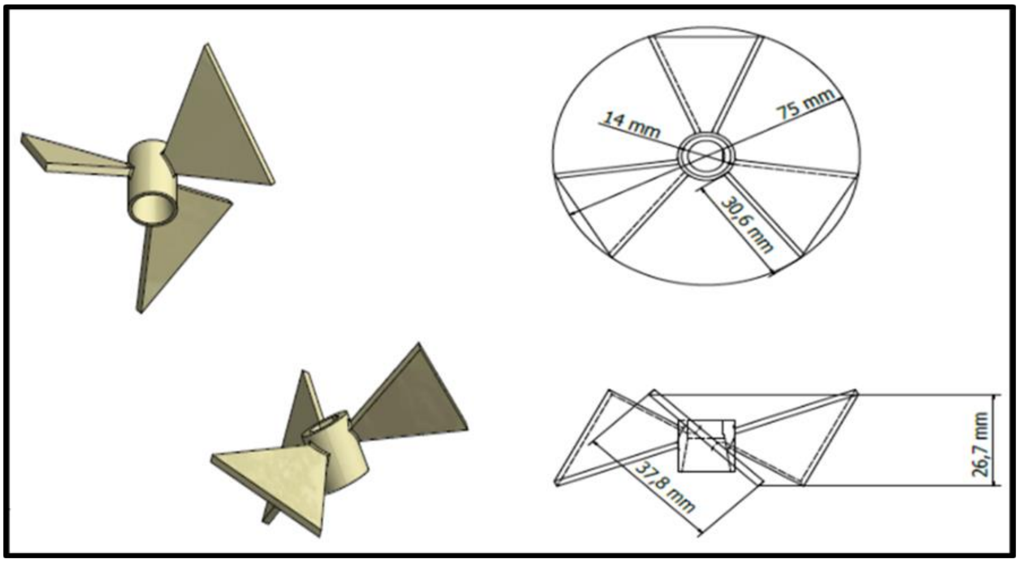


Figure S2 Design and dimensions of butterfly impeller

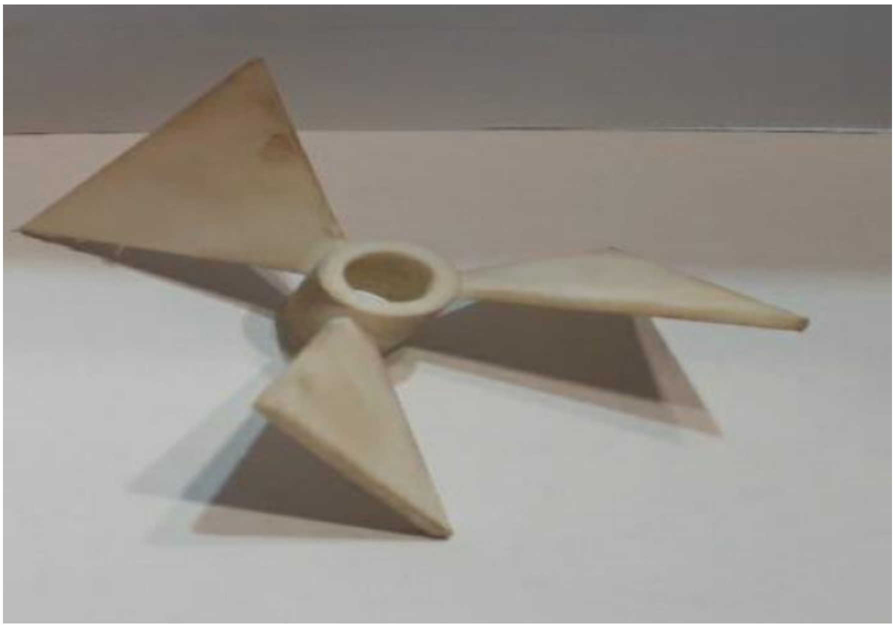

Figure S3 Butterfly impeller as was used in our experiments

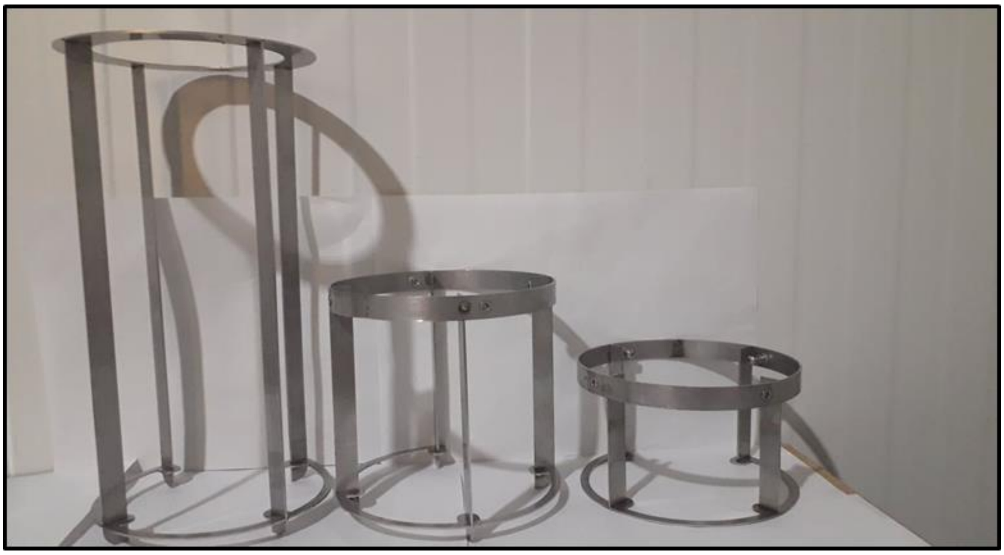

Figure S4 Three different baffles used in this study, Left: FB, Middle: HB, Right: SB
